# Enhanced biodegradation of n-hexane by *Pseudomonas* sp. strain NEE2

**DOI:** 10.1038/s41598-019-52661-0

**Published:** 2019-11-12

**Authors:** Shanying He, Yaoqi Ni, Li Lu, Qiwei Chai, Haiyang Liu, Chunping Yang

**Affiliations:** 1 Zhejiang Provincial Key Laboratory of Solid Waste Treatment and Recycling, College of Environmental Science and Engineering, Zhejiang Gongshang University, Hangzhou, Zhejiang 310018 China; 2 Guangdong Provincial Key Laboratory of Petrochemical Pollution Processes and Control, School of Environmental Science and Engineering, Guangdong University of Petrochemical Technology, Maoming, Guangdong 525000 China; 3 Datang Environment Industry Group Co., Ltd, Beijing, 100097 China; 4 College of Environmental Science and Engineering, Hunan University and Key Laboratory of Environmental Biology and Pollution Control (Hunan University), Ministry of Education, Changsha, Hunan 410082 China

**Keywords:** Pollution remediation, Atmospheric chemistry

## Abstract

*Pseudomonas* sp. strain NEE2 isolated from oil-polluted soils could biodegrade n-hexane effectively. In this study, the secretory product of n-hexane biodegradation by NEE2 was extracted, characterized, and investigated on the secretory product’s enhanced effect on n-hexane removal. The effects of various biodegradation conditions on n-hexane removal by NEE2, including nitrogen source, pH value, and temperature were also investigated. Results showed that the secretory product lowered surface tension of water from 72 to 40 mN/m, with a critical micelle concentration of 340 mg/L, demonstrating that there existed biosurfactants in the secretory product. The secretory product at 50 mg/L enhanced n-hexane removal by 144.4% within 48 h than the control group. The optimum conditions for n-hexane removal by NEE2 were at temperature of 25–30 °C, pH value of 7–8, and (NH_4_)_2_SO_4_ as nitrogen source. Besides n-hexane, NEE2 could also utilize a variety of carbon sources. These results proved that NEE2 can consume hydrophobic volatile organic compounds (VOCs) to produce biosurfactants which can further enhance hydrophobic VOCs degradation.

## Introduction

Emission of volatile organic compounds (VOCs) has been a major concern because of their negative impact and damage to human health, ecosystems, and regional climate^1^. VOC emissions constitute approximately 7% of those of all atmospheric pollutants^2^. VOCs are the organic compounds with high saturated vapor pressure, low boiling point, low molecular weight and low volatilization at normal temperature, such as volatile polycyclic aromatic hydrocarbons (PAHs), amines, halogenated hydrocarbons, alcohols, esters, aldehydes, ketones, ethers, acids, and low-boiling hydrocarbon^3^. There are natural and anthropogenic emissions. Anthropogenic emissions are from processes including crude oil production, solvent applications in chemical production, resin industry, paint production, wax industry, and automobile exhaust^4^.

At present, various technologies have been established to remove VOCs, for example, condensation^5^, adsorption^6^, absorption^7^, combustion^8^, membrane separation^9^, and biological treatment^10–12^. Although advanced oxidation processes^13–15^ and photocatalysis^16–18^ have been successfully applied for the removal of various organic compounds, biological treatment systems are commercially competitive for VOC removal because of their advantages includeing low-energy consumption, low operation costs, and avoidance of secondary pollution^19^. Cai *et al*.^20^ found that the removal efficiency of methyl isobutyl ketone in a trickle-bed air biofilter exceeded 99%. Biological treatment systems perform well in the treatment of hydrophilic VOCs. However, they are often poorly effective for the removal of hydrophobic VOCs (e.g., toluene, hexane, ethene, ethane, methane, pentane, acetylene, cyclohexane, etc.), because of their the substrate’s low solubility or availability in water^3,21^. Estrada *et al*.^22^ compared the performances of different treatment techniques in removing highly, medially and lowly hydrophobic compounds, and found that biological filtration, activated sludge, biological trickling, chemical washing and incineration performed well in the treatment of low hydrophobic compounds, and the removal efficiency of techniques reached 99%. In the treatment of the highly hydrophobic compound, the removal efficiency of biological filtration, activated sludge, and biological trickling performed not well. The results indicated that the biological method was effective to remove hydrophilic VOCs but not hydrophobic VOCs.

Many methods have been investigated for enhancing the bioavailability of hydrophobic VOCs in biological treatment systems, including innovative bioreactors, biofiltration with pretreatment, low-moisture operation, the application of specialized microbes, the utilization of hydrophilic VOCs and the addition of surfactants^3,23–25^. Two-liquid-phase bioreactor using silicone oil and water performed better than the traditional one-liquid-phase biofilter in removal efficiency and fluctuation resistance capability for hydrophobic VOC removal^26^. UV pretreatment and ozone oxidation were also applied for better removal performance for hydrophobic VOC removal in subsequent biofilters^27,28^; Sun *et al*.^29^ treated 10 kinds of VOCs at 150~200 ppm in air with a UV reactor and found that the VOCs and their products were mineralized in off-gas absorption solutions. Rene *et al*.^30^ applied the fungus *Sporothrix variecibatus* to treat gas-phase styrene and acetone mixtures in a biofilter, and the total elimination capacity was as high as 360 gm^−3^ h^−1^, with nearly 97.5% removal of styrene and 75.6% for acetone. *Sporothrix variecibatus* biofilter for gas-phase styrene treatment also performend well^30^.

Surfactants have been widely used in wastewater treatment and soil remediation based on their ability to increase solubility, lower surface tensions, wetting ability and foaming capacity^31,32^. In recent years, in the removal of hydrophobic VOCs in biological treatment systems, chemical surfactants have been introduced to reduce the surface tension and form micelles for enhancing the bioavailability of hydrophobic VOCs^3,33–36^. Compared with chemical surfactants, biosurfactants are more promising because they are more environmentally friendly, less toxic and more biodegradable^32,37^. Moreover, a few studies have been reported on the use of waste gas by microorganisms to produce biosurfactants for promoting waste gas degradation. For example, Cheng *et al*.^38^ isolated a strain of *Pseudomonas fluorescens* PT in the process of removing α-pinene (moderately hydrophilic VOC) in the biotrickling filter and found that it can produce a surface-active substance during its metabolism. Li *et al*.^39^ reported that in the process of exploring the biotrickling filter to degrade octamethylcyclotetrasiloxane (D4), *Pseudomonas aeruginosa* S240 produced rhamnolipids which were found to be the main factor for improving D4 removal. Tu *et al*.^40^ reported that saponins (a kind of biosurfactants) could effectively enhance n-hexane removal and decrease the biomass accumulation rate in a biotrickling filter, demonstrating that the use of biosurfactants can improve the hydrophobic VOCs removal efficiency in biological treatment systems. However, minimal reports are available on the utilization of hydrophobic VOCs by microorganisms to produce biosurfactants for promoting the degradation of hydrophobic VOCs.

n-Hexane is produced and consumed as a solvent. Large amounts of n-Hexane has been emitted to the environment^41^. Long-term exposure to n-hexane gas in the production environment can damage human’s nerve system^42^. n-Hexane has lower solubility than aromatic compounds (e.g. toluene)^43^. However, up to now, there is no report about whether microorganisms can use n-hexane to produce biosurfactants and improve n-hexane removal efficiency.

In our previous study, a bacteria strain that can biodegrade n-hexane has been isolated from oil-polluted soils, and identified to be *Pseudomonas* sp. strain NEE2 (simplified as NEE2). The 16S rDNA sequence obtained from *Pseudomonas* sp. strain NEE2 was deposited in the National Center for Biotechnology Information database with the accession number MK429838, and preserved in the China Center for Type Culture Collection (Wuhan, China). The removal efficiency for 50 μL (132 mg/L) n-hexane by NEE2 on Day 2 exceeded 60%, and reached more than 70% on Day 5. It was found that NEE2 was in logarithmic growth in 24–48 hours when the amount of n-hexane added was 100–200 μL.

The objective of this study was to extract and characterize from the secretory product of NEE2, investigate the effect of surfactant addition, nitrogen source, pH, and temperature on the n-hexane removal by NEE2, and evaluate the removal effect of NEE2 on other carbon sources, so as to provide a new method for the removal of hydrophobic VOCs.

## Results

### Extraction and characterization of the secretory product of NEE2

**Figure 1:**
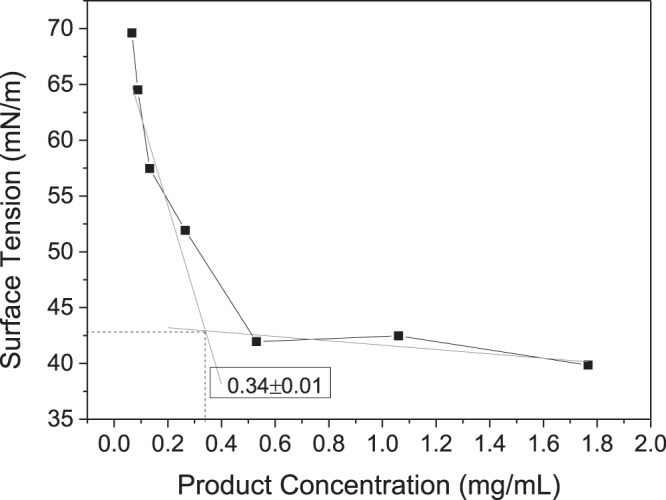
Relationship between surface tension and concentration of the secretory product.

As shown in Fig. 1, the secretory product of NEE2 reduced the surface tension of water from 72 to 40 mN/m, indicating that the secretory product has surface tension lowering activity and the bacteria NEE2 has the ability of producing biosurfactant.

Critical micelle concentration (CMC) was always used as a measure of the surface activity of a surfactant. The Fig. 1 showed that the secretory product has a CMC value of 340 mg/L, indicating further that the secretory product of NEE2 has the characteristics of surfactant.

### Effects of surfactant addition on n-hexane removal by NEE2

**Figure 2:**
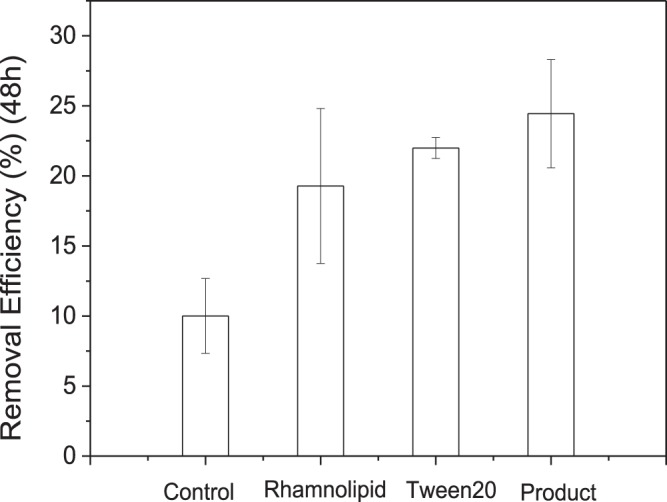
Effects of the addition of the secretory product on n-hexane removal by *Pseudomonas* sp. strain NEE2.

To further verify the surfactant property of the secretory product of NEE2, the degradation abilities of the secretory product extracted from NEE2 biodegradation, Tween20 and rhamnolipid on the n-hexane were compared (Fig. 2), and the results showed that all the addition treatment had significant effects on the n-hexane removal within 48 h (p < 0.05). Compared with the control group, n-hexane removal by the secretory product extracted from NEE2 biodegradation, rhamnolipid and Tween addition, increased by 144.4%, 92.8% and 120.0%, respectively. Therefore, it could be deduced that NEE2 produced surfactants which improved the n-hexane removal.

### Effects of nitrogen sources, pH value and temperature on n-hexane removal by NEE2

#### Nitrogen sources

Nutrients may be limiting factors that affect microbial degradation of pollutants. In this study, n-hexane was a hydrophobic VOC, and its solubility in water at room temperature was only 0.014%. Nevertheless, the dosage of added nitrogen sources were set at 1.2 g/L, and all these nitrogen sources were aqueous solutions, thus the influence of nitrogen sources on the solubility of n-hexane could be ignored.

**Figure 3:**
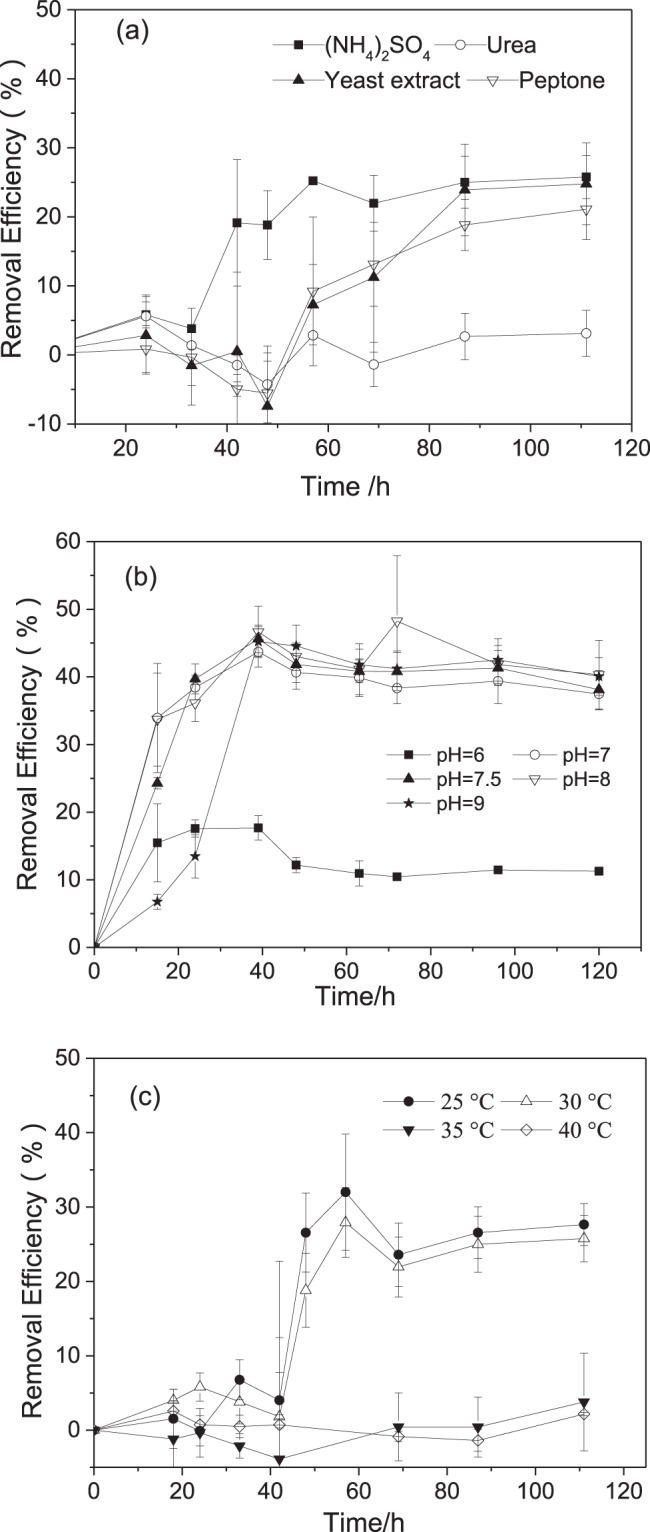
Effects of nitrogen sources, pH and temperature on n-hexane removal by *Pseudomonas* sp. strain NEE2. (**a**) Nitrogen sources; (**b**) pH; (**c**) Temperature.

As shown in Table 1, when the nitrogen source was ammonium sulfate, yeast extract and peptone, NEE2 grew well, while NEE2 did not grow in the medium with urea. Figure 3a also showed that the n-hexane was not degraded when the nitrogen source was urea. These results indicated that urea is unsuitable for NEE2. Within 33 hours, there was no significant difference of n-hexane removal efficiency between the (NH_4_)_2_SO_4,_ yeast extract and peptone, but after that, the degradation rate of n-hexane in (NH_4_)_2_SO_4_ was found to be significantly higher than that of yeast extract and peptone, suggesting that (NH_4_)_2_SO_4_ was more suitable as the nitrogen source for strain NEE2.

**Table 1:** The growth of *Pseudomonas* sp. strain NEE2 for n-hexane degradation in different nitrogen sources, pH and temperature.

Nitrogen sources	Results	Temperature/°C	Results	pH	Results
(NH_4_)_2_SO_4_	+	25	+	6	+
Urea	−	30	+	7	+
Yeast extract	+	35	−	7.5	+
Peptone	+	40	−	8	+
				9	+

+, Positive growth; −, no growth.

### pH

pH may influence the bacterial physiological and biochemical processes, the activity of enzymes, and the solubility of pollutants, which result in an effect on the pollutant degradation^44^.

Table 1 and Fig. 3b showed that NEE2 could grow and degrade n-hexane at pH 6~9. However, the n-hexane removal at pH 6 was only 15%. As the pH value increased to 7 and 8, n-hexane removal efficiency significantly improved and reached 43.7%~46.6% at 39 h. Although the removal efficiency at pH 9 reached 45.2% at the 39th hour, it was obviously lower than that at pH 7 and 8 and even lower than that at pH 6 during the initial 24 hours, indicating that the alkali conditions had a greater negative effect on n-hexane removal.

**Figure 4:**
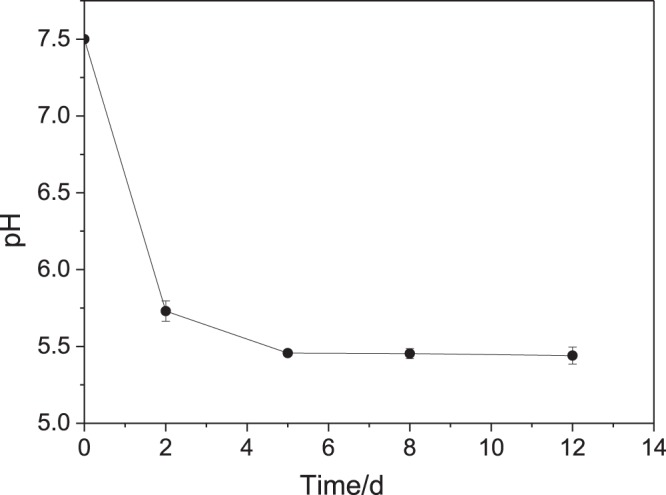
Relationship of pH of the fermentation broth and time during the biodegradation of n-hexane by *Pseudomonas* sp. strain NEE2.

During the bacterial growth, the pH of the culture solution decreased significantly (Fig. 4), indicating that NEE2 metabolized n-hexane and produced acid material. In addition, after 39 hours, the removal efficiency of all groups did not increase any more, indicating that some factors such as the accumulation of acid product limited the n-hexane removal. Leson *et al*.^45^ found that the biodegradation of pollutants may produce acidic by-products such as oxidation of sulfur/nitrogen compounds and chlorinated organic compounds^45^. Therefore, in practical applications, a chemical buffer such as lime would be required to prevent the decrease of pH.

### Temperature

Temperature plays an important role in the biodegradation of pollutants by directly affecting the chemical nature of the contaminants and the physiological/biochemical characteristics of the microflora^46^. In addition, temperature also affects the solubility of contaminants, which affects microbial degradation^47^. Proper temperature will promote gas dissolution for optimal microbial catabolism.

Table 1 showed that the temperature of 25–30 °C was suitable for NEE2 to grow, while at the temperature higher than 35 °C, the strain can not grow (Table 1) and degrade n-hexane (Fig. 3c). Therefore, in practical applications, NEE2 is unsuitable for the reactors treating the exhaust gases at high temperature.

### Removal of other carbon sources by NEE2

In general, biofilters are only suitable for removing VOCs with medium to low Henry’s phase equilibrium constant^3^. For VOCs with high Henry coefficient, i.e. hydrophobic VOCs, the removal capacity is limited^21^. Mass transfer of volatile organic compounds is a key factor that affects the performance of bioreactor^10^. Since NEE2 can produce biosurfactant, which may improve the mass transfer^38,39^, examination of other carbon sources removal by NEE2 was investigated, and the results showed that NEE2 could use a variety of carbon sources such as hydrophobic VOCs, non-volatile alkanes or hydrophilic VOC as a sole carbon to grow, while it did not utilize the polycyclic aromatic hydrocarbon (Table 2). Although the growth of NEE2 in the o-chlorodiphenyl was not as well as in other VOC treatment such as n-hexane, isooctane, xylene and acetone, NEE2 was able to utilize o-chlorodiphenyl at a certain concentration to grow (Table 2), indicating that NEE2 can utilize chlorinated hydrocarbon, but its utilization is limited.

**Table 2:** The growth of *Pseudomonas* sp. strain NEE2 for n-hexane degradation in different carbon sources.

Type of hydrocarbon	Hydrocarbon	Results	Remarks
Control	/	−	
Hydrophobic VOCs	n-Hexane	++	Alkane
	Isooctane	++	Alkane
	Xylene	++	Monoaromatic hydrocarbon
	o-Dichlorobenzene	+	Chlorinated hydrocarbon
Non-volatile alkanes	n-Tanane	++	
	Dodecane	++	
	Tetradecane	++	
	n-Hexadecane	++	
Hydrophilic VOC	Acetone	++	
Polyaromatic hydrocarbons	Phenanthrene	−	
	Anthracene	−	

++, +, Positive growth; −, no growth.

## Discussion

Because of the surface activity of biosurfactants, the surface tension of the liquid can be reduced. According to this principle, surface tension is an important parameter that is commonly used to evaluate the effectiveness of a biosurfactant^48^, and a low surface tension indicates that the surface activity is strong. The decrease of the surface tension and CMC value of the secretory products (Fig. 1) proved that NEE2 could biodegrade n-hexane and produce biosurfactants simultaneously. Similarly, Cheng *et al*.^38^ isolated a strain of *Pseudomonas fluorescens* PT in the process of removing α-pinene in the biotrickling filter, and found that it could produce a surface-active substance during its metabolism. Li *et al*.^39^ reported that *Pseudomonas aeruginosa* S240 produced rhamnolipids which were found to be the main factor for improving D4 removal. In these studies, α-pinene is a moderately hydrophilic VOC and D4 is a type of volatile methyl siloxane. However, n-Hexane is a hydrophobic VOC, which has lower solubility, higher toxicity to bacterial species and is more harmful to human. The result in this study proved that it is possible for the utilization of hydrophobic VOCs by microorganisms to produce biosurfactants.

According to the biofilm theory proposed by Ottengraf^49^, the biological method for removing gas pollutants such as VOCs needs three steps: at first, gaseous VOCs are transferred to a liquid phase; then, VOCs in the liquid phase are captured and absorbed by the microorganisms; at last, VOCs enter the microbes and are biodegraded and even completely mineralized into carbon dioxide, H_2_O and other non-toxic small molecules. On the basis of this theory, a lot of investigations on the effects of surfactants on the enhanced degradation of hydrophobic VOCs were carried out including those on the enhanced gas-liquid mass transfer at the presence of surfactants^3,35^; increased hydrophobicity of the cell surface for better biosorption and absorption^50^; and improved biodegradation due to the surfactant-activation of some key enzymes taking part in the biodegradation^51^.

In conclusion, the secretory product extracted from NEE2 biodegradation has surface tension lowering activity, and the secretory product contains surfactants. The decrease of the surface tension and CMC value of the secretory product proved that NEE2 could biodegrade n-hexane and produce biosurfactants simultaneously. The result demonstrated that it is possible for the utilization of hydrophobic VOCs by microorganisms to produce biosurfactants. The addition of secretory product has a significant effect on the n-hexane removal within 48 h. NEE2 could use n-hexane to produce biosurfactants and thus promoting the degradation of n-hexane. NEE2 could utilize a variety of carbon sources, including hydrophilic VOC, hydrophobic VOCs, and non-volatile alkanes. Therefore, NEE2 has a potential in applications to the biodegradation of the heterogeneous organic pollutants.

## Materials and Methods

### Bacterial culture media

n-Hexane (C_6_H_14_, chemically purity of 97%) was used as the target contaminant. The medium YP was used to culture microorganisms. Medium YP contained (g/L): KH_2_PO_4_, 0.4; K_2_HPO_4_, 0.4; MgSO_4_ 7H_2_O, 0.3; and (NH_4_)_2_SO_4_, 1.2. Five milliliters of sterilized solution I was added to 1 L of the medium YP. The solution I contained (g/L): ZnSO_4_ 7H_2_O, 0.5; FeSO_4_ 7H_2_O, 1; CaCl_2_, 4; and MnSO_4_ H_2_O, 0.16.

The autoclaved medium FL was used for examination of other carbon sources removal by NEE2. Medium FL contained (g/L): (NH_4_)_2_SO_4_, 6; KH_2_PO_4_, 3; Na_2_HPO_4_, 2; MgSO_4_, 0.5; NaCl, 1.0; and agar, 20.

### Extraction of secretory product

The bacteria strain NEE2 was cultured in 250 mL bottles containing 50 mL of medium YP and 0.2 mL n-hexane as the sole carbon source for 36–48 h and mixed with 120 rpm at 30 °C. Then, the culture was centrifuged at 8000 × g and 4 °C for 10 min for cell collection. The collected cells were re-suspended using ultra-pure water and the concentration of the re-suspended cells was measured. The re-suspended cells were transferred to 9 bottles (250 mL) containing 50 mL fresh medium YP, and the initial bacteria concentration in each bottle was the same by controlling the addition volume of the re-suspended cells^35^. The concentrations of bacteria were determined by measuring the optical density at 600 nm (OD_600_) by using a UV-1900 UV spectrophotometer (Persee, Beijing, China). Next, 1000 μL hexane was injected into each bottle. All bottles were cultivated in the shaking incubator at 30 °C and 120 rpm. After cultivation for 12 days, cells were removed from the culture broth by centrifugation at 8000 × g for 10 min. This operation was repeated for two times. Referring to the method by Nitschke *et al*.^52^ and Raza *et al*.^53^, the secretory product was isolated from cell-free broth by acid precipitation after adjusting the broth pH to 2.0 using 6 M H_2_SO_4_^52,53^. Cell-free broth was concentrated by evaporation in a 50 °C water bath, and extracted 3 times with ethyl acetate. The organic phases were collected and evaporated using a Termovap Sample Concentrator. The separated product was dissolved in the ultrapure water, and extracted with dichloromethane for 3 times. Finally, the organic phase evaporated with a Termovap Sample Concentrator to get the production. The 450 mL NEE2 biodegradation solution after 12-day cultivating was extracted to obtain light brown solid production, which was measured to be 5.3 mg.

### Measurement of critical micelle concentration

Critical micelle concentration of the secretory product were determined by measuring the surface tension^54^. The secretory product formulated into solutions of different concentration rations: the 5.3 mg production was serial diluted in water of 3, 5, 10, 20, 40, 60, and 80 mL, and the surface tension of each solution was measured for 3 times. The surface tension of the solution was measured by a platinum-plate-tensiometer (Shanghai Fangrui Instrument Co., Shanghai, China) at room temperature.

### Effects of surfactant addition on n-hexane removal by NEE2

To further verify the characteristics of the secretory product, the effect of the addition of secretory product, Tween 20 (chemical surfactant) and rhamnolipid (biosurfactant) on the n-hexane removal was investigated, respectively. The concentration of surfactants was set as 50 mg/L.

The secretory product of NEE2 was dissolved in ultrapure water to prepare a 0.5 g/L surfactant solution. Similarly, Tween20 and rhamnolipid were also formulated to 0.5 g/L surfactant solutions. The bacteria NEE2 was cultured and re-suspended as previously described. The re-suspended cells were transferred to 250-mL bottles containing 45 mL fresh Medium YP, and ensure the initial bacteria concentration in each bottle was the same by controlling the addition volume of the re-suspended cells. Next, 5 mL surfactant solution (0.5 g/L) (after filter sterilization or autoclaving) was injected into each bottle, to prepare a culture with 50 mg/L surfactant^33,40^. The bottles without surfactant addition was set as the control group, and bottles with no bacteria inoculation were used as the blank group. All groups were set in triplicate. Finally, bottles were sealed with butyl rubber and an aluminum cap, and 100 μL (264 mg/L) hexane was injected into each bottle. All bottles were cultivated in the shaking incubator at 30 °C and 120 rpm. The n-Hexane concentration in the headspace of each bottle was measured at the 48th hour.

### Effects of nitrogen sources, pH and temperature on n-hexane removal by NEE2

(NH_4_)_2_SO_4,_ urea, peptone, and yeast extract at 1.2 g/L were selected as the nitrogen sources. The bacteria NEE2 was cultured and re-suspended as previously stated. Next, the bottle was sealed with butyl rubber and an aluminum cap^41^. Then, 100 μL (264 mg/L) hexane was injected into each bottle. All bottles were cultivated in the shaking incubator at 30 °C and 120 rpm. Under the same condition, bottles with no bacteria inoculation were used as the blank group. All experiments were performed in triplicate. The n-Hexane concentration in the headspace of each bottle was measured at 24, 33, 42, 48, 57, 69, 87, and 111 h respectively.

The effects of pH and the temperature on n-hexane removal were investigated. In the pH and temperature experiments, (NH_4_)_2_SO_4_ was used as nitrogen source, and experiments were similarly operated as previously stated. For the temperature experiments, the bottles were maintained at 25, 30, 35, 40 °C respectively, the removal efficiency of n-hexane in each bottle was assayed. For the pH experiments, the pH was set to 6, 7, 7.5, 8 and 9 respectively and the n-hexane concentration were measured.

Since the measurement of the n-hexane concentration required maintaining the tightness of bottles, an experiment was additionally set to monitor the pH of the solution. Fifteen bottles were divided into five groups, and the pH of the solution in each group was measured on the 1st, 2nd, 5th, 8th and 12th day, respectively.

### Removal of other carbon sources by NEE2

The ability of NEE2 to degrade carbon sources besides n-hexane was evaluated. The tested carbon sources included hydrophilic VOC (acetone), hydrophobic VOCs (alkane: n-hexane and isooctane; monoaromatic hydrocarbon: xylene; chlorinated hydrocarbon: o-dichlorobenzene), non-volatile alkanes (n-tanane, dodecane, tetradecane and n-hexadecane) and polyaromatic hydrocarbons (phenanthrene and anthracene). The bacteria NEE2 was cultured in 250 mL bottles for 36–48 h at 120 rpm and 30 °C. Each bottle contains 50 mL of medium YP with 0.2 mL n-hexane as the sole carbon source. Then, the 200 μL culture was spread on medium FL plate supplemented with 10 or 20 μL of the test hydrocarbon as a sole carbon and energy source. Plates without hydrocarbon were used as the control. Samples were cultured in an incubator at 30 °C. NEE2 cells were inoculated to a 250-ml bottle containing 50 ml of mediumYP supplemented with 10 or 20 mg of a polyaromatic hydrocarbon phenanthrene or anthracene, and cultured in a shaking incubator at 120 rpm and 30 °C. All the samples were cultured for 7 days before analysis.

### Analytical methods

The concentration of n-hexane was analyzed by using gas chromatograph (7890 A, Agilent, USA) equipped with a flame ionization detector (FID) and a capillary column type HP-5 (30 m × 0.320 mm; 2.25 μm film, Agilent, USA). The temperatures of the oven, injector, and detector were 80 °C, 200 °C, and 200 °C, respectively. Purified air and hydrogen were applied at a flow rate of 450 mL min^−1^ and 40 mL min^−1^ respectively. High-purity nitrogen gas was used as the carrier gas at a flow rate of 30 ml min^−1^. Removal efficiencies (RE, %) were estimated by the following equation:

1 $$RE=\frac{{C}_{B}-{C}_{A}}{{C}_{B}}\times 100$$

where C_B_ and C_A_ represent the n-hexane concentrations of the blank and experimental groups, respectively.

### Statistical analysis

All the results have been presented as mean ± standard deviation (S.D). A one-way analysis of variance (ANOVA) by SPSS statistical software was used. P-values less than 0.05 were considered to represent statistically significant difference.

### Ethical statement

This article does not contain any studies with human participants or animals performed by any of the authors.
